# Disparities in Under-Five Child Injury Mortality between Developing and Developed Countries: 1990–2013

**DOI:** 10.3390/ijerph13070653

**Published:** 2016-07-07

**Authors:** Yun Huang, Yue Wu, David C. Schwebel, Liang Zhou, Guoqing Hu

**Affiliations:** 1 Department of Environmental and Occupational Health, Xiangya School of Public Health, Central South University, 110 Xiangya Rd., Changsha 410078, China; 803110@csu.edu.cn (Y.H.); wuyue7802@csu.edu.cn (Y.W.); 2 Department of Psychology, University of Alabama at Birmingham, Birmingham, AL 35233, USA; schwebel@uab.edu; 3 Department of Social Medicine and Health Management, Xiangya School of Public Health, Central South University, 110 Xiangya Rd., Changsha 410078, China; 203156@csu.edu.cn; 4 Department of Epidemiology and Health Statistics, Xiangya School of Public Health, Central South University, 110 Xiangya Rd., Changsha 410078, China

**Keywords:** child, injury, mortality, global

## Abstract

*Objective*: Using estimates from the 2013 Global Burden of Disease (GBD) study, we update evidence on disparities in under-five child injury mortality between developing and developed countries from 1990 to 2013. *Methods*: Mortality rates were accessed through the online visualization tool by the GBD study 2013 group. We calculated percent change in child injury mortality rates between 1990 and 2013. Data analysis was conducted separately for <1 year and 1–4 years to specify age differences in rate changes. *Results*: Between 1990 and 2013, over 3-fold mortality gaps were observed between developing countries and developed countries for both age groups in the study time period. Similar decreases in injury rates were observed for developed and developing countries (<1 year: −50% vs. −50% respectively; 1–4 years: −56% vs. −58%). Differences in injury mortality changes during 1990–2013 between developing and developed nations varied with injury cause. There were greater reductions in mortality from transport injury, falls, poisoning, adverse effects of medical treatment, exposure to forces of nature, and collective violence and legal intervention in developed countries, whereas there were larger decreases in mortality from drowning, exposure to mechanical forces, and animal contact in developing countries. Country-specific analysis showed large variations across countries for both injury mortality and changes in injury mortality between 1990 and 2013. *Conclusions*: Sustained higher child injury mortality during 1990–2013 for developing countries merits the attention of the global injury prevention community. Countries that have high injury mortality can benefit from the success of other countries.

## 1. Introduction

Injuries are a major killer of children worldwide. Every day over 2000 children and teenagers die from an injury, and more than 95% of all child injury deaths occur in developing countries [1].

The United Nations Millennium Development Goal 4 (MDG 4) set the goal “to reduce mortality of children under 5 years by two-thirds between 1990 and 2015” [2]. Given the significant disparity in injury mortality between developing countries and developed countries, accomplishing that goal will require significant reduction of injury prevalence in developing countries [2]. Although some research considers global progress of MDG 4 and the trends in injury mortality across nations [3,4,5,6,7,8,9,10,11,12,13], no published studies offer trends specifically focused on global injury mortality gaps between developing countries and developed countries among children under age 5.

A challenge to pediatric injury prevention globally is the need to tailor interventions to the developmental stage of the child [14]. Among the most significant developmental stages is the transition from infancy to toddlerhood. Injury risks to infants under age 1, who are not yet mobile and suffer injuries largely due to parental negligence or abuse, are very different from injury risks to toddlers and preschoolers ages 1–4, who are mobile, curious, and exploratory [15].

Thus, this study was designed to examine the disparities in injury mortality among children under age 5 in developing and developed countries since 1990 with consideration of differences between infants under age 1 vs. toddlers and preschoolers ages 1–4. We used the online data visualization tool Global Burden of Disease (GBD) Compare to conduct our analysis. Three specific research questions were evaluated: (a) did developing countries experience the same changes in under-five injury mortality as developed countries between 1990 and 2013? (b) did age- and cause-specific child injury mortality rates change equally in the study time period? and (c) within developing countries and within developed countries, did all countries witness equal changes in child injury mortality rate?

## 2. Methods

### 2.1. Ethics Statement

This study was a secondary analysis of publicly-available anonymized aggregate data and was approved by the Medical Ethics Committee of Central South University, China (XYGW-2015-05).

### 2.2. Data Source

Mortality data come from the estimates of the 2013 Global Burden of Disease Study (GBD 2013) which include cause-, sex-, age-, region and country-specific estimates of injuries between 1990 and 2013. Based on data from vital registration systems, verbal autopsy studies, and other sources, the GBD 2013 study group uses a comprehensive approach to estimate age-specific mortality (including 95% uncertain interval), which is composed of estimation of under-5 mortality, estimation of adult mortality, and estimation of age-specific mortality. Details of the methodology, including sophisticated algorithms to cope with rare event outcomes and countries with small populations, are published elsewhere [10,11,16]. The estimates of GBD 2013 can be accessed through the online data visualization tool at [17].

### 2.3. Data Analysis

The GBD 2013 group divided the 187 countries of the world into categories of 50 developed countries and 137 developing countries based on development status; we followed that division [18]. Given the developmental differences among the age groups and suggestions from Bhalla et al. [15], we considered injury mortality separately for children <1 year and 1–4 years.

Based on the International Classification of Diseases (ICD), the GBD 2013 study group divided injuries into 14 categories: (1) road injury; (2) other transport injury; (3) falls; (4) drowning; (5) fire, heat and hot substances; (6) poisonings; (7) exposure to mechanical forces; (8) adverse effects of medical treatment; (9) animal contact; (10) unintentional injuries not classified elsewhere; (11) forces of nature; (12) self-harm; (13) interpersonal violence; and (14) collective violence and legal intervention. The first 11 categories are unintentional and the last 3 categories are intentional.

We used percent change in mortality rates to measure changes in mortality between 1990 and 2013, which was calculated as “((the rate in 2013−the rate in 1990)/the rate in 1990) × 100%”. Cause-specific trends in mortality rates were plotted by age group and country income.

## 3. Results

**Figure 1:**
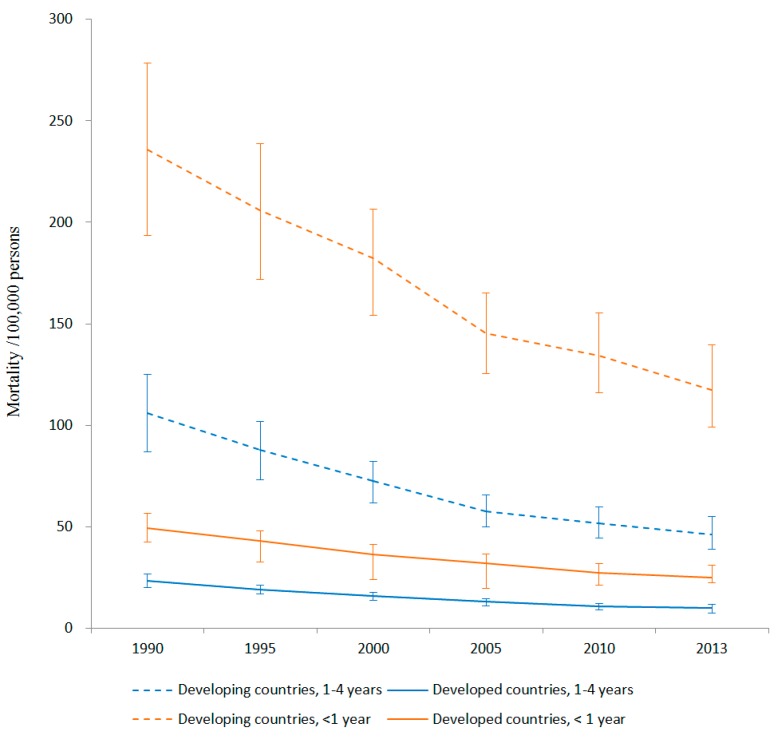
Global under-five child injury mortality per 100,000 persons by age and country income, 1990–2013.

Between 1990 and 2013, global deaths from injuries decreased from 282,711 to 145,420 for children <1 year and from 483,433 to 221,701 for children 1–4 years. For the entire duration of 1990–2013, injury mortality rates were notably higher in developing countries compared to developed countries for both the <1 year and the 1–4 years age groups (Figure 1).

Our first primary question was whether developing countries experienced the same changes in young children’s injury mortality that developed countries experienced. We found that injury mortality declined substantially from 1990 to 2013 for both age groups in both developed and developing countries, and at similar rates (<1 year: −50% vs. −50%; 1–4 years: −56% vs. −58%).

**Figure 2:**
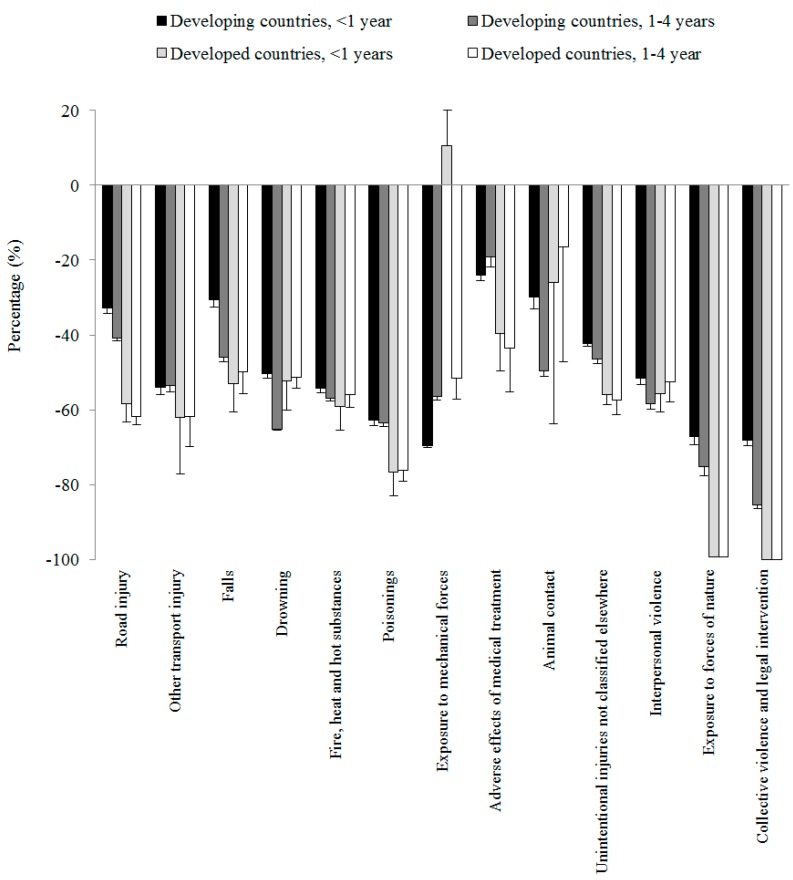
Percent change in under-five injury mortality between 1990 and 2013 by age, cause, and country income.

Our second question was whether age- and cause-specific child injury mortality rates changed equally in developing and developed countries. Subgroup analysis by cause, age group and country income produced diverse results: (1) greater reductions in deaths from road traffic crashes, other transport injury, falls, poisoning, adverse effects of medical treatment, exposure to forces of nature, and collective violence and legal intervention in developed countries compared to developing countries; (2) less reductions in deaths from drowning, exposure to mechanical forces, and animal contact in developed countries compared to developing countries; (3) an increase in injury mortality from exposure to mechanical forces for under-1 children in developed countries; and (4) nearly equal decreases in deaths from all other injury causes in both types of countries (Table 1, Figure 2).

**Figure 3:**
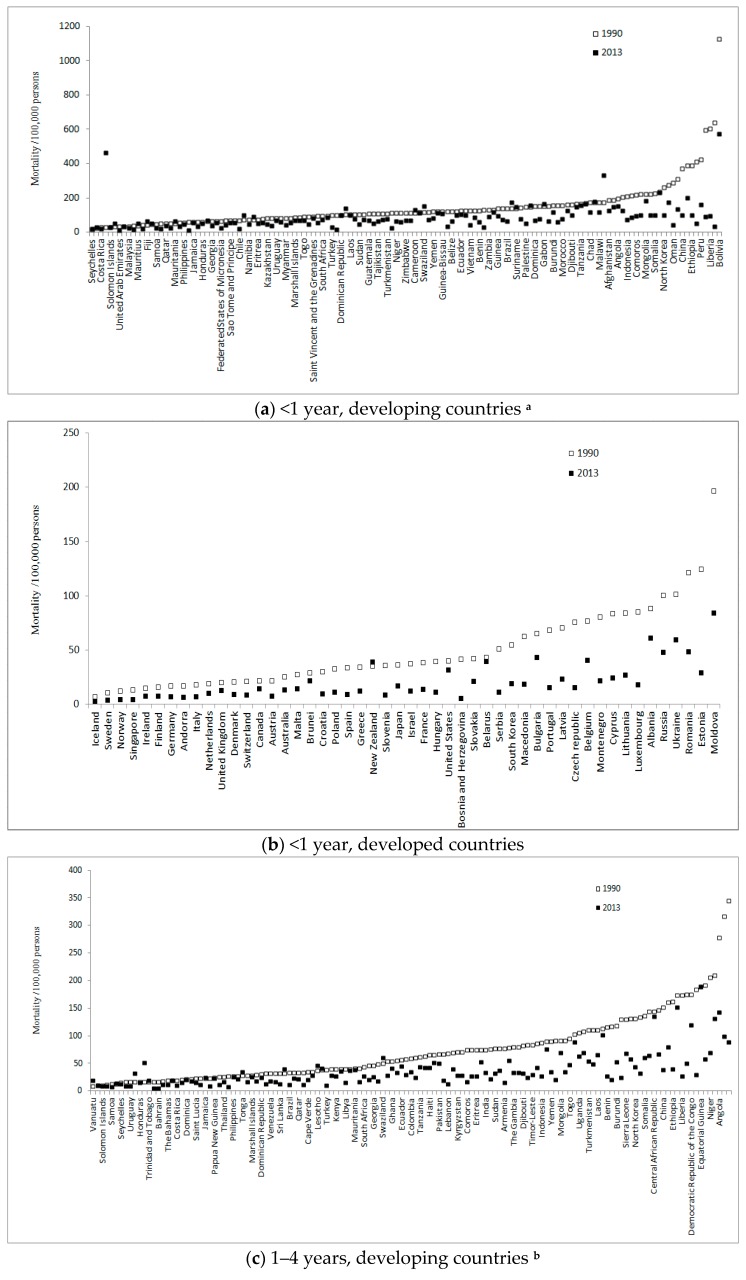

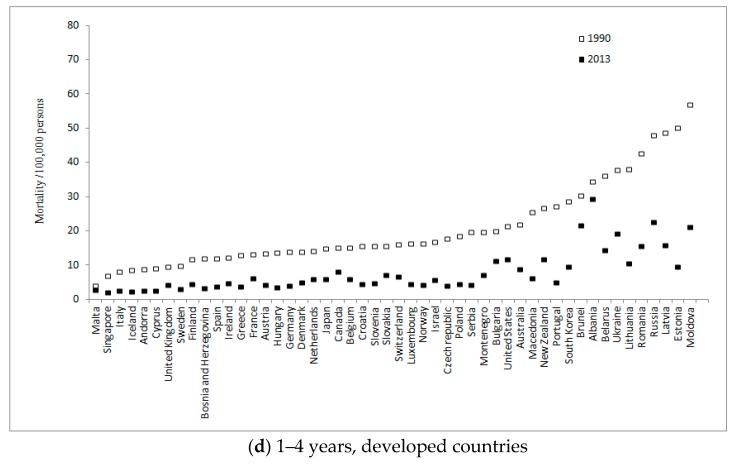
Injury mortality for children under 5 in 187 countries between 1990 and 2013. **^a^** Country labels ranked by mortality of 1990 in Figure 3a: Seychelles, Taiwan, Costa Rica, Syria, Solomon Islands, Vanuatu, United Arab Emirates, Egypt, Malaysia, Bahrain, Mauritius, Cuba, Fiji, Antigua and Barbuda, Samoa, Kuwait, Qatar, Thailand, Mauritania, Libya, Philippines, Saudi Arabia, Jamaica, The Bahamas, Honduras, Trinidad and Tobago, Georgia, Panama, Federated States of Micronesia, Grenada, Sao Tome and Principe, Paraguay, Chile, Kiribati, Namibia, Papua New Guinea, Eritrea, Barbados, Kazakhstan, Jordan, Uruguay, Saint Lucia, Myanmar, Senegal, Marshall Islands, Nicaragua, Togo, Azerbaijan, Saint Vincent and the Grenadines, Venezuela, South Africa, Ghana, Turkey, Sri Lanka, Dominican Republic, Lesotho, Laos, Cambodia, Sudan, Botswana, Guatemala, Madagascar, Tajikistan, Cape Verde, Turkmenistan, Armenia, Niger, Mexico, Zimbabwe, Timor-Leste, Cameroon, Tonga, Swaziland, The Gambia, Yemen, Congo, Guinea-Bissau, Tunisia, Belize, Uzbekistan, Ecuador, Kenya, Vietnam, Mali, Benin, El Salvador, Zambia, Cote d'Ivoire, Guinea, Iraq, Brazil, Guyana, Suriname, Argentina, Palestine, Central African Republic, Dominica, Colombia, Gabon, Nepal, Burundi, Algeria, Morocco, Democratic Republic of the Congo, Djibouti, Uganda, Tanzania, Haiti, Chad, Nigeria, Malawi, Equatorial Guinea, Afghanistan, Sierra Leone, Angola, Burkina Faso, Indonesia, Kyrgyzstan, Comoros, Bangladesh, Mongolia, Mozambique, Somalia, Pakistan, North Korea, India, Oman, Rwanda, China, Bhutan, Ethiopia, Iran, Peru, Maldives, Liberia, Lebanon, Bolivia. **^b^** Country labels ranked by mortality of 1990 in Figure 3(**c**): Vanuatu, Mauritius, Solomon Islands, Barbados, Samoa, Antigua and Barbuda, Seychelles, Malaysia, Uruguay, Fiji, Honduras, Syria, Trinidad and Tobago, Cuba, Bahrain, Grenada, The Bahamas, Paraguay, Costa Rica, Egypt, Dominica, Taiwan, Saint Lucia, Kuwait, Jamaica, Chile, Papua New Guinea, Federated States of Micronesia, Thailand, United Arab Emirates, Philippines, Saint Vincent and the Grenadines, Tonga, Argentina, Marshall Islands, Belize, Dominican Republic, Sao Tome and Principe, Venezuela, Mexico, Sri Lanka, Kiribati, Brazil, Guatemala, Qatar, El Salvador, Cape Verde, Panama, Lesotho, Guyana, Turkey, Botswana, Kenya, Zimbabwe, Libya, Suriname, Mauritania, Nicaragua, South Africa, Palestine, Georgia, Azerbaijan, Swaziland, Namibia, Ghana, Cambodia, Ecuador, Iraq, Colombia, Myanmar, Tanzania, Kazakhstan, Haiti, Congo, Pakistan, Madagascar, Lebanon, Jordan, Kyrgyzstan, Morocco, Comoros, Algeria, Eritrea, Uzbekistan, India, Tunisia, Sudan, Tajikistan, Armenia, Cote d'Ivoire, The Gambia, Peru, Djibouti, Nepal, Timor-Leste, Zambia, Indonesia, Gabon, Yemen, Saudi Arabia, Mongolia, Senegal, Togo, Cameroon, Uganda, Guinea-Bissau, Turkmenistan, Vietnam, Laos, Chad, Benin, Oman, Burundi, Maldives, Sierra Leone, Guinea, North Korea, Mozambique, Somalia, Malawi, Central African Republic, Burkina Faso, China, Mali, Ethiopia, Nigeria, Liberia, Rwanda, Democratic Republic of the Congo, Iran, Equatorial Guinea, Bhutan, Niger, Afghanistan, Angola, Bolivia, Bangladesh.

Our third question was whether countries experienced equal changes in young children’s injury mortality rate within the classifications of developing and developed countries. In general, country-specific analysis revealed similar gaps in both injury mortality and changes in mortality between developed countries and developing countries for both age groups between 1990 and 2013 (Figure 3a–d). However, great variations were observed within developed countries and within developing countries.

In 2013, the largest gap in injury mortality among children under age 1 reached 61-fold in developing countries (Saudi Arabia, 9.4 per 100,000 persons vs. Bolivia, 572.6 per 100,000 persons) and 31-fold in developed countries (Iceland, 2.7 per 100,000 person vs. Moldova, 84.2 per 100,000 persons) (Figure 3a,b).

Among children aged 1–4 years, we discovered up to 33-fold differences between Bahrain (5.8 per 100,000 persons) and Equatorial Guinea (189.3 per 100,000 persons) in developing countries, and 15-fold mortality gaps between Singapore (2.0 per 100,000 persons) and Albania (29.3 per 100,000 persons) in developed countries (Figure 3c,d).

Except for an 11% increase in under-1 injury mortality in New Zealand, the injury mortality rates displayed substantial or slight declines for both age groups in all 49 developed countries (Figure 3b). In contrast, 11 developing countries experienced 20% or greater rises in under-1 injury mortality: Syria (1440%), Equatorial Guinea (89%), Vanuatu (59%), Fiji (52%), Kiribati (41%), Lesotho (36%), Swaziland (32%), Mauritius (32%), Guyana (25%), Mauritania (21%) and Papua New Guinea (21%) (Figure 3a).

Eight developing countries witnessed over 10% increases in injury mortality for children ages 1–4 years: Syria (203%), Vanuatu (98%), Fiji (93%), Lesotho (25%), Tonga (24%), Swaziland (21%), Kiribati (21%), and Trinidad and Tobago (10%) (Figure 3c). In contrast, injury mortality for children ages 1–4 years decreased in all 50 developed countries (Figure 3d).

Country-specific injury mortality data and percent change in rates between 1990 and 2013 for developing countries are provided as supplemental materials.

## 4. Discussion

The child injury rate in both developed and developing countries declined between 1990 and 2013. The decline rates in developing countries were similar to those in developed countries, but child injury mortality rates in developing countries remain significantly higher in developing countries for both the <1 year and the 1–4 year age groups. Implications of these findings for public health are transparent: more efforts are needed to reduce high child injury mortality, especially in developing countries. Although cultural context must be considered, many interventions focused on preventing injuries to young children living in developing nations might be modeled after those successfully implemented in developed nations. As examples, improvements in laws mandating child safety seat use and measures to reduce alcohol-impaired driving have contributed to significant decline in motor vehicle traffic–related deaths in the United States [19]. Similar measures may be adapted to local context and are likely to be successful in developing countries [1]. We found diverse changes in cause-specific injuries between developed countries and developing countries and among the two developmental stages studied. These results may reflect the effects of many factors, including enhanced injury prevention efforts in particular domains, social-economic development and its influence on injury risk in particular countries, social upheaval in countries and natural disasters.

As others have reported, we found substantial disparities in child injury mortality rates both within the group of developed countries and within the group of developing countries [20,21]. These variations may reflect various influences, including globalization and urbanization, motorization, environmental change in particular countries, and governmental efforts to injury control.

Our results yielded a few unexpected results. One was the increase in injuries due to exposure to mechanical force among children under age 1 in developed countries. We are unsure what to speculate may have caused this result. Also surprising was the increase in mortality among children under age 1 in particular countries. *Post-hoc* analyses uncovered a range of causes in different countries and regions. Two extremely high increases are noteworthy: (a) a 343% increase in unintentional suffocation in children ages 28–364 days in New Zealand (from 5.0 to 22.0/100,000 persons); and (b) a large rise in deaths from collective violence and legal intervention (from 0.0 to 127.1/100,000 persons in children ages 28–364 days) in Syria [17]. The finding from Syria is likely veridical given the violence present in that nation in 2013; the result from New Zealand may be an artifact of data collection or coding issues, or it may be veridical. We generally conclude there is need for context-specific future investigation of increases in injury rates in particular countries and regions that evaluate possible causes.

Globalization eases dissemination of ideas and knowledge about injury prevention [22], but it also leads to increased transport in places where road safety management is weak [23], unplanned and ill-resourced urbanization, and high risk of motor vehicle crashes [24]. In the last two decades, many developing countries have experienced rapid urbanization. For example, the proportion of urban inhabitants in China rose from 26% in 1990 to 50% in 2010 [25].

Rapid motorization, which often accompanies urbanization, leads to shifts in primary transport for citizens. In particular, motorization increases the exposure of inhabitants to road traffic crashes, especially in places where road traffic control far lags behind motorization process. Many of these crashes involve vulnerable road users, who are exposed to motorized traffic at higher rates as a nation or community motorizes. The motorization that accompanied the urbanization of China offers an example, road traffic mortalities in China increased from 3.9/100,000 persons in 1985 to 7.6/100,000 persons in 2005 [26].

Another factor that contributes to disparities in child injury rates across countries is how governments take efforts to control injuries. Most fatal and non-fatal child injuries are preventable [1], and many effective interventions are known. Unfortunately, those effective interventions are not always implemented, especially in developing nations, due to economic, technical, or political factors [1,20,27,28,29,30].

The findings have important implications. First, it suggests encouraging evidence toward the goals of MDG 4 [2], indicating incremental success in controlling young children’s injuries globally. At the same time, however, the sustained disparities between developing countries and developed countries are striking and indicate the need to prioritize injury control in developing countries, which lags. In particular, several countries suffered significant rises in child injury mortality between 1990 and 2013. Efforts to control child injury in these nations may be particular priorities and might require support from government and non-profit agencies [31].

Our analysis is limited somewhat by the scarcity of high-quality data from low- and middle-income countries, as detailed in GBD publications [5,10,11,16]. Also a limitation is the fact that the data do not allow us to explore causal reasons behind decreases in global child injury mortality and large disparities in child injury between developing countries and developed countries. Finally, we focused only on linear trends between 1990 and 2013. It is likely that nonlinear trends occurred in particular countries. Future research might consider case-by-case or detailed analyses that explore nonlinear trends in injury rates over time as well as potential causes of the data patterns we detected.

## 5. Conclusions

We conclude that the under-five child injury mortality disparity between developed countries and developing countries has persisted from 1990 to 2013, despite nearly equal and significant injury mortality reductions in both groups of countries. Addressing the lagging injury control in developing countries should be a priority of the global injury prevention community.

## Figures and Tables

**Table 1:** Change in cause-specific injury mortality (/100,000 persons) among under-five children between 1990 and 2013.

Age Group/Development Status/Cause	1990		2013		Percent Change in Rate (95% CI)
	Deaths	Rate	Deaths	Rate	
**<1 year, developing countries**					
Road injury	22,983	19.7	15,984	13.2	−32.9 (−34.2, −31.5)
Other transport injury	5538	4.8	2647	2.2	−53.9 (−56.0, −51.7)
Falls	12,630	10.8	9074	7.5	−30.7 (−32.5, −28.8)
Drowning	19,069	16.4	9814	8.1	−50.3 (−51.5, −49.1)
Fire, heat and hot substances	18,443	15.8	8732	7.2	−54.3 (−55.5, −53.1)
Poisonings	7362	6.3	2842	2.4	−62.8 (−64.3, −61.1)
Exposure to mechanical forces	72,460	62.2	22,843	18.9	−69.6 (−70.0, −69.1)
Adverse effects of medical treatment	19,306	16.6	15,211	12.6	−24.0 (−25.6, −22.4)
Animal contact	4957	4.3	3598	3.0	−30.0 (−32.9, −26.9)
Unintentional injuries not classified elsewhere	70,392	60.4	42,014	34.8	−42.4 (−43.1, −41.7)
Interpersonal violence	10,663	9.2	5350	4.4	−51.6 (−53.2, −50.0)
Exposure to forces of nature	3106	2.7	1061	0.9	−67.0 (−69.3, −64.7)
Collective violence and legal intervention	7902	6.8	2610	2.2	−68.1 (−69.5, −66.7)
**<1 year, developed countries**					
Road injury	884	5.5	338	2.3	−58.4 (−63.2, −52.8)
Other transport injury	59	0.4	21	0.1	−61.2 (−76.4, −36.2)
Falls	397	2.5	172	1.2	−52.8 (−60.5, −43.6)
Drowning	383	2.4	168	1.1	−52.2 (−60.2, −42.7)
Fire, heat and hot substances	502	3.1	189	1.3	−59.0 (−65.3, −51.5)
Poisonings	223	1.4	48	0.3	−76.6 (−82.8, −68.0)
Exposure to mechanical forces	788	4.9	801	5.5	10.7 (0.3, 22.1)
Adverse effects of medical treatment	327	2.0	181	1.2	−39.7 (−49.7, −27.7)
Animal contact	19	0.1	13	0.1	−25.5 (−63.2, −50.9)
Unintentional injuries not classified elsewhere	3253	20.4	1319	9.0	−55.8 (−58.6, −52.9)
Interpersonal violence	964	6.0	392	2.7	−55.7 (−60.6, −50.2)
Exposure to forces of nature	47	0.3	0	0.0	-
Collective violence and legal intervention	53	0.3	0	0.0	-
**1–4 years, developing countries**					
Road injury	81,125	18.4	50,530	10.8	−40.9 (−41.6, −40.3)
Other transport injury	7514	1.7	3687	0.8	−53.5 (−55.2, −51.6)
Falls	25,687	5.8	14,620	3.1	−46.0 (−47.1, −44.9)
Drowning	192,313	43.5	70,478	15.1	−65.2 (−65.5, −64.9)
Fire, heat and hot substances	48,500	11.0	21,991	4.7	−57.0 (−57.7, −56.3)
Poisonings	19,327	4.4	7432	1.6	−63.5 (−64.5, −62.5)
Exposure to mechanical forces	27,890	6.3	12,777	2.7	−56.5 (−57.4, −55.6)
Adverse effects of medical treatment	7082	1.6	6037	1.3	−19.1 (−21.9, −16.3)
Animal contact	12,584	2.8	6684	1.4	−49.6 (−51.1, −48.1)
Unintentional injuries not classified elsewhere	27,302	6.2	15,397	3.3	−46.5 (−47.5, −45.4)
Interpersonal violence	11,107	2.5	4874	1.0	−58.4 (−59.7, −56.9)
Exposure to forces of nature	1683	0.4	440	0.1	−75.2 (−77.7, −72.5)
Collective violence and legal intervention	5880	1.3	908	0.2	−85.4 (−86.3, −84.3)
**1–4 years, developed countries**					
Road injury	4242	6.5	1449	2.5	−61.9 (−64.1, −59.5)
Other transport injury	271	0.4	92	0.2	−62.1 (−70.1, −52.0)
Falls	852	1.3	382	0.7	−49.9 (−55.6, −43.5)
Drowning	3210	4.9	1398	2.4	−51.4 (−54.3, −48.2)
Fire, heat and hot substances	2007	3.1	792	1.4	−55.9 (−59.4, −52.2)
Poisonings	1389	2.1	296	0.5	−76.2 (−79.0, −73.0)
Exposure to mechanical forces	805	1.2	350	0.6	−51.4 (−57.2, −45.0)
Adverse effects of medical treatment	212	0.3	107	0.2	−43.6 (−55.3, −28.9)
Animal contact	41	0.1	30	0.1	−18.3 (−49.0, −30.9)
Unintentional injuries not classified elsewhere	1525	2.3	581	1.0	−57.5 (−61.3, −53.2)
Interpersonal violence	866	1.3	368	0.6	−52.5 (−58.0, −46.4)
Exposure to forces of nature	9	0.0	0	0.0	-
Collective violence and legal intervention	11	0.0	0	0.0	-

Notes: 95% CI: 95% confidence interval based on Poisson regression; -: Percent change in rates was not calculated due to zero value of mortality rates at one or both time points.

## Supplementary Materials

The following are available online at www.mdpi.com/1660-4601/13/7/653/s1, Table S1: Child injury mortality in developing countries by nation (<1 year, 1990 vs. 2013), Table S2: Injury mortality in developing countries by nation (1–4 years, 1990 vs. 2013).
