# Supplementary material for: Disparities in Under-Five Child Injury Mortality between Developing and Developed Countries: 1990–2013
Source: Int J Environ Res Public Health. 2016 Jul 7;13(7):653. doi: 10.3390/ijerph13070653 (PMC4962194; doi:10.3390/ijerph13070653)
Supplement: Supplementary file 1 [file ijerph-13-00653-s001.pdf]

Supplementary Materials: Disparities in Under-Five Child Injury Mortality between Developing and Developed Countries: 1990–2013

Yun Huang, Yue Wu, David C. Schwebel, Liang Zhou and Guoqing Hu

Table S1. Child injury mortality in developing countries by nation (<1 year, 1990 vs. 2013).

Country	1990	2013	% Change in Rates
Seychelles	15.5	18.5	19
Taiwan	26.6	29.3	10
Costa Rica	28.2	20.6	−27
Syria	30.0	461.9	1440
Solomon Islands	30.7	29.2	−5
Vanuatu	31.3	49.8	59
United Arab Emirates	33.5	10.6	−68
Egypt	33.5	32.3	−4
Malaysia	34.6	24.6	−29
Bahrain	37.4	16.0	−57
Mauritius	38.7	50.9	32
Cuba	41.9	21.6	−48
Fiji	42.9	65.0	52
Antigua and Barbuda	47.6	49.5	4
Samoa	47.7	22.9	−52
Kuwait	49.8	19.6	−61
Qatar	51.0	36.8	−28
Thailand	52.5	24.6	−53
Mauritania	53.0	64.1	21
Libya	53.8	31.0	−42
Philippines	56.0	45.6	−19
Saudi Arabia	57.9	9.4	−84
Jamaica	58.8	54.8	−7
The Bahamas	59.3	34.9	−41
Honduras	60.5	51.2	−15
Trinidad and Tobago	62.9	66.6	6
Georgia	64.0	37.4	−42
Panama	64.0	56.9	−11
Federated States of Micronesia	65.6	25.8	−61
Grenada	67.7	42.2	−38
Sao Tome and Principe	67.8	55.6	−18
Paraguay	68.5	56.0	−18
Chile	70.5	21.0	−70
Kiribati	71.3	100.5	41
Namibia	72.0	47.6	−34
Papua New Guinea	73.2	88.5	21
Eritrea	73.3	52.0	−29
Barbados	75.1	56.0	−25
Kazakhstan	80.8	47.9	−41
Jordan	82.5	38.0	−54
Uruguay	83.2	67.5	−19
Saint Lucia	83.2	57.7	−31

Table S1. *Cont.*

Country	1990	2013	% Change in Rates
Myanmar	83.4	43.5	−48
Senegal	83.5	53.6	−36
Marshall Islands	87.2	69.9	−20
Nicaragua	87.5	68.7	−22
Togo	88.5	67.9	−23
Azerbaijan	89.8	45.3	−50
Saint Vincent and the Grenadines	91.5	83.7	−9
Venezuela	93.1	57.3	−39
South Africa	94.6	72.6	−23
Ghana	96.7	84.3	−13
Turkey	97.0	27.6	−72
Sri Lanka	97.8	14.5	−85
Dominican Republic	97.9	97.4	0
Lesotho	101.9	138.1	36
Laos	103.0	97.3	−6
Cambodia	104.3	78.7	−24
Sudan	104.4	44.6	−57
Botswana	105.0	73.3	−30
Guatemala	107.4	67.4	−37
Madagascar	107.8	52.3	−52
Tajikistan	108.0	61.9	−43
Cape Verde	108.7	71.1	−35
Turkmenistan	109.1	78.2	−28
Armenia	111.6	25.7	−77
Niger	112.5	63.1	−44
Mexico	113.1	58.2	−49
Zimbabwe	113.6	69.9	−39
Timor-Leste	113.7	68.0	−40
Cameroon	114.0	129.4	14
Tonga	115.0	110.9	−4
Swaziland	115.2	152.3	32
The Gambia	115.9	71.8	−38
Yemen	119.0	83.7	−30
Congo	119.6	110.5	−8
Guinea-Bissau	120.1	105.7	−12
Tunisia	121.5	34.7	−71
Belize	121.5	64.3	−47
Uzbekistan	122.9	100.4	−18
Ecuador	125.1	104.8	−16
Kenya	125.4	99.3	−21
Vietnam	125.6	40.6	−68
Mali	125.9	87.0	−31
Benin	127.2	58.8	−54
El Salvador	128.4	29.0	−77
Zambia	131.7	90.5	−31
Cote d'Ivoire	131.8	116.2	−12
Guinea	137.0	95.3	−30
Iraq	137.8	71.2	−48
Brazil	139.5	63.5	−55
Guyana	139.9	174.9	25
Suriname	140.3	149.5	7
Argentina	142.9	76.4	−47
Palestine	147.1	50.4	−66
Central African Republic	148.4	154.5	4

Table S1. *Cont.*

Country	1990	2013	% Change in Rates
Dominica	149.7	69.2	−54
Colombia	151.1	76.4	−49
Gabon	152.2	164.2	8
Nepal	153.4	63.1	−59
Burundi	154.6	115.3	−25
Algeria	156.5	58.6	−63
Morocco	156.9	75.3	−52
Democratic Republic of the Congo	160.2	126.8	−21
Djibouti	161.3	99.8	−38
Uganda	163.8	149.0	−9
Tanzania	164.5	156.3	−5
Haiti	171.5	166.2	−3
Chad	172.5	116.4	−33
Nigeria	174.6	180.3	3
Malawi	175.0	117.1	−33
Equatorial Guinea	175.1	331.2	89
Afghanistan	186.3	126.2	−32
Sierra Leone	186.4	145.8	−22
Angola	195.6	151.7	−22
Burkina Faso	203.8	125.5	−38
Indonesia	210.3	74.2	−65
Kyrgyzstan	212.1	84.2	−60
Comoros	216.1	96.8	−55
Bangladesh	222.1	100.8	−55
Mongolia	222.1	182.6	−18
Mozambique	224.2	96.9	−57
Somalia	227.8	97.3	−57
Pakistan	234.0	230.3	−2
North Korea	260.0	99.9	−62
India	275.4	175.7	−36
Oman	287.1	40.5	−86
Rwanda	309.6	136.3	−56
China	372.2	101.0	−73
Bhutan	388.5	201.4	−48
Ethiopia	388.9	99.7	−74
Iran	412.3	50.7	−88
Peru	424.3	162.3	−62
Maldives	596.4	89.5	−85
Liberia	602.7	96.1	−84
Lebanon	638.0	33.9	−95
Bolivia	1126.7	572.6	−49

Table S2. Injury mortality in developing countries by nation (1–4 years, 1990 vs. 2013).

Country	1990	2013	% Change in Rates
Vanuatu	9.6	19.0	98
Mauritius	10.2	10.9	7
Solomon Islands	10.3	9.8	−6
Barbados	12.2	9.4	−23
Samoa	13.1	7.8	−41
Antigua and Barbuda	13.4	13.9	4
Seychelles	16.0	13.4	−16
Malaysia	16.5	9.5	−43
Uruguay	16.6	8.5	−49
Fiji	16.9	32.6	93
Honduras	17.0	16.1	−5
Syria	17.2	51.9	203
Trinidad and Tobago	17.4	19.2	10
Cuba	17.5	5.8	−67
Bahrain	17.5	5.8	−67
Grenada	18.6	12.2	−34
The Bahamas	19.8	11.6	−41
Paraguay	19.9	19.3	−3
Costa Rica	20.1	10.7	−47
Egypt	21.3	16.1	−25
Dominica	21.6	20.8	−4
Taiwan	22.3	17.9	−20
Saint Lucia	22.7	16.1	−29
Kuwait	23.4	11.3	−52
Jamaica	23.6	24.3	3
Chile	23.9	8.5	−64
Papua New Guinea	24.5	23.6	−3
Federated States of Micronesia	25.5	11.6	−55
Thailand	26.5	17.5	−34
United Arab Emirates	26.8	8.0	−70
Philippines	27.5	25.9	−6
Saint Vincent and the Grenadines	27.7	22.3	−19
Tonga	27.9	34.6	24
Argentina	28.4	17.0	−40
Marshall Islands	28.8	25.9	−10
Belize	29.6	17.7	−40
Dominican Republic	30.8	24.4	−21
Sao Tome and Principe	31.7	13.5	−58
Venezuela	32.0	17.7	−45
Mexico	32.1	17.0	−47
Sri Lanka	32.3	12.6	−61
Kiribati	32.7	39.4	21
Brazil	33.3	12.1	−64
Guatemala	33.5	23.7	−29
Qatar	33.6	21.5	−36
El Salvador	34.1	11.1	−67
Cape Verde	34.4	21.2	−39
Panama	35.4	28.8	−19
Lesotho	37.4	46.8	25
Guyana	38.4	40.7	6
Turkey	38.9	10.7	−72
Botswana	39.9	29.1	−27
Kenya	39.9	26.8	−33
Zimbabwe	39.9	36.7	−8

Table S2. *Cont.*

Country	1990	2013	% Change in Rates
Libya	40.1	15.5	−62
Suriname	40.2	38.0	−6
Mauritania	41.4	38.8	−6
Nicaragua	41.6	16.4	−61
South Africa	44.1	27.3	−38
Palestine	45.9	21.1	−54
Georgia	46.8	25.8	−45
Azerbaijan	49.5	17.6	−64
Swaziland	50.4	61.0	21
Namibia	53.7	28.2	−47
Ghana	54.4	41.1	−24
Cambodia	55.0	33.1	−40
Ecuador	57.4	45.0	−21
Iraq	57.8	29.6	−49
Colombia	59.0	34.3	−42
Myanmar	61.0	25.0	−59
Tanzania	62.3	43.9	−30
Kazakhstan	63.8	42.5	−33
Haiti	65.7	43.0	−35
Congo	66.4	51.7	−22
Pakistan	66.6	50.8	−24
Madagascar	66.8	19.2	−71
Lebanon	68.0	13.0	−81
Jordan	69.3	40.1	−42
Kyrgyzstan	70.5	27.9	−60
Morocco	70.6	28.7	−59
Comoros	74.2	16.3	−78
Algeria	74.4	27.4	−63
Eritrea	74.8	27.0	−64
Uzbekistan	75.0	52.8	−30
India	75.0	33.3	−56
Tunisia	75.6	22.6	−70
Sudan	76.9	32.9	−57
Tajikistan	77.4	37.0	−52
Armenia	77.6	15.5	−80
Cote d'Ivoire	79.1	55.5	−30
The Gambia	79.3	33.0	−58
Peru	79.5	34.2	−57
Djibouti	82.2	31.9	−61
Nepal	83.6	24.8	−70
Timor-Leste	83.7	30.2	−64
Zambia	87.0	43.2	−50
Indonesia	87.2	26.8	−69
Gabon	89.9	76.5	−15
Yemen	90.3	35.0	−61
Saudi Arabia	91.5	20.3	−78
Mongolia	91.5	69.7	−24
Senegal	91.6	34.6	−62
Togo	95.4	47.9	−50
Cameroon	103.4	89.2	−14
Uganda	105.2	62.6	−40
Guinea-Bissau	108.4	69.5	−36
Turkmenistan	110.5	54.6	−51
Vietnam	111.1	48.9	−56

Table S2. *Cont.*

Country	1990	2013	% Change in Rates
Laos	111.1	66.0	−41
Chad	114.1	102.2	−10
Benin	115.7	27.1	−77
Oman	116.8	20.5	−82
Burundi	118.8	52.8	−56
Maldives	130.0	24.6	−81
Sierra Leone	130.8	68.8	−47
Guinea	131.5	58.6	−55
North Korea	131.6	43.5	−67
Mozambique	134.6	32.1	−76
Somalia	136.3	60.0	−56
Malawi	144.3	65.0	−55
Central African Republic	144.3	135.6	−6
Burkina Faso	147.4	66.7	−55
China	152.1	39.3	−74
Mali	160.5	79.8	−50
Ethiopia	161.7	40.6	−75
Nigeria	174.0	152.4	−12
Liberia	174.4	26.8	−85
Rwanda	174.7	49.9	−71
Democratic Republic of the Congo	175.2	120.4	−31
Iran	184.1	29.4	−84
Equatorial Guinea	188.8	189.3	0
Bhutan	191.4	57.6	−70
Niger	205.8	70.0	−66
Afghanistan	210.1	131.3	−38
Angola	278.8	143.5	−49
Bolivia	316.5	99.8	−68
Bangladesh	345.0	88.6	−74

© 2016 by the authors; licensee MDPI, Basel, Switzerland. This article is an open access article distributed under the terms and conditions of the Creative Commons by Attribution (CC-BY) license (http://creativecommons.org/licenses/by/4.0/).
